# Critical Diagnosis and Management of a Case of a Partial Molar Cesarean Scar Ectopic Pregnancy

**DOI:** 10.7759/cureus.79009

**Published:** 2025-02-14

**Authors:** Samuel R Raine, Vera Schulte, Jess Floyd, Kylie Culp

**Affiliations:** 1 Obstetrics and Gynecology, University of Colorado Anschutz Medical Campus, Aurora, USA; 2 Obstetrics and Gynecology, Denver Health and Hospitals, Denver, USA

**Keywords:** cesarean scar ectopic, first-trimester ultrasound, gravid hysterectomy, molar pregnancy, sterilization

## Abstract

The objective of this case report is to highlight critical findings of a cesarean scar partial molar ectopic pregnancy and review the counseling and management strategies. A 43-year-old G3P0201 patient with a history of two cesarean deliveries at 10 weeks 3 days by last menstrual period presented to the ED with a week of dark brown discharge. The patient had previously been diagnosed with a pregnancy of uncertain viability five weeks prior at an outside institution but was lost to follow-up. Transvaginal ultrasound and pelvic computed tomography were performed to guide counseling and recommendations for the management of what was concerning for both a partial molar pregnancy and a cesarean scar ectopic pregnancy (CSEP). After counseling, the patient underwent a gravid total abdominal hysterectomy and bilateral salpingectomy for definitive management of the CSEP, as she had satisfied her fertility goals. Pathology of the specimen confirmed a partial molar pregnancy in the cesarean scar without evidence of an accreta. Close review of early pregnancy imaging is critical in making a diagnosis of a caesarean scar ectopic pregnancy complicated by a molar pregnancy. A gravid hysterectomy may reduce the morbidity of this combination of rare pregnancy pathologies.

## Introduction

We present a rare case of a cesarean scar ectopic pregnancy (CSEP) with abnormal features consistent with a partial molar pregnancy. A CSEP occurs when the early pregnancy implants into the site of a cesarean scar [1-3]. The site of implantation may be classified as either intrinsic, where the implantation occurs inside the uterine scar niche, or extrinsic, where the pregnancy implants on top of the uterine scar [1,2]. CSEP has become more prevalent as a diagnosis, as the number of cesarean deliveries has increased and more literature has been published highlighting the pathology [1]. The incidence is currently reported as approximately 1:1800 to 1:2600 pregnancies; however, it is suggested by many that the pathology is underdiagnosed [1-3]. Both with early intervention and expectant management, a common morbidity with the management of CSEP is maternal hemorrhage [1-4]. This risk for adverse maternal morbidity may be complicated when a CSEP has additional pathologies that increase the risk of maternal hemorrhage at a time of management, such as a molar pregnancy [1,2].

Our unique case emphasizes the importance of elevated suspicion when reviewing early pregnancy imaging to offer early intervention for life-threatening pregnancies such as CSEP alongside a molar pregnancy.

## Case presentation

A 43-year-old G3P0201 patient with a history of two cesarean deliveries at approximately 10 weeks 3 days by an uncertain last menstrual period presented to the ED with a chief complaint of one week of brown discharge. Approximately one month prior, the patient was evaluated at an outside ED for five days of vaginal bleeding and was diagnosed with an intrauterine pregnancy with a fetal pole without cardiac activity. At the initial outside ED encounter, the patient was informed that while the pregnancy did not have cardiac activity, its viability was uncertain based on the early gestational age. The patient was counseled to get follow-up care within two weeks; however, due to socioeconomic barriers to care, the patient was unable to access follow-up before the representation in the ED a month later. On review of these outside records, no documentation of CSEP concern or partial molar pregnancy was noted.

**Figure 1:**
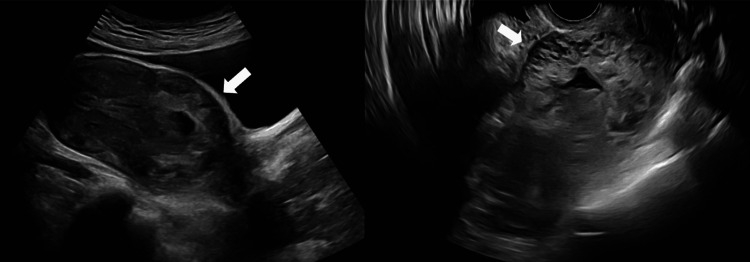
Transabdominal and transvaginal ultrasound images at the time of representation, indicating a low-lying gestational sac concerning for cesarean scar ectopic pregnancy. The arrow highlights the distinct uterine outpouching toward the bladder, which is pathognomonic of cesarean scar ectopic pregnancies. The hyperemic appearance around the gestational sac had significant vascularity, which was noted. These findings heightened suspicion for a partial molar pregnancy.

As part of the workup to evaluate the brown discharge in the setting of a pregnancy of uncertain viability, a transvaginal ultrasound identified a 7.9 x 6.1 x 6 cm hyperemic, heterogenous mass within the lower uterine segment with minimal overlying myometrium and a small fetal pole without cardiac activity corresponding to approximately six weeks gestation (Figure 1). Initial quantitative beta-human chorionic gonadotropin (b-hCG) was 47,855 mIU/mL. These imaging findings were consistent with the outside institution’s ultrasound a month prior when the b-hCG level was 78,424 mIU/mL. 

**Figure 2:**
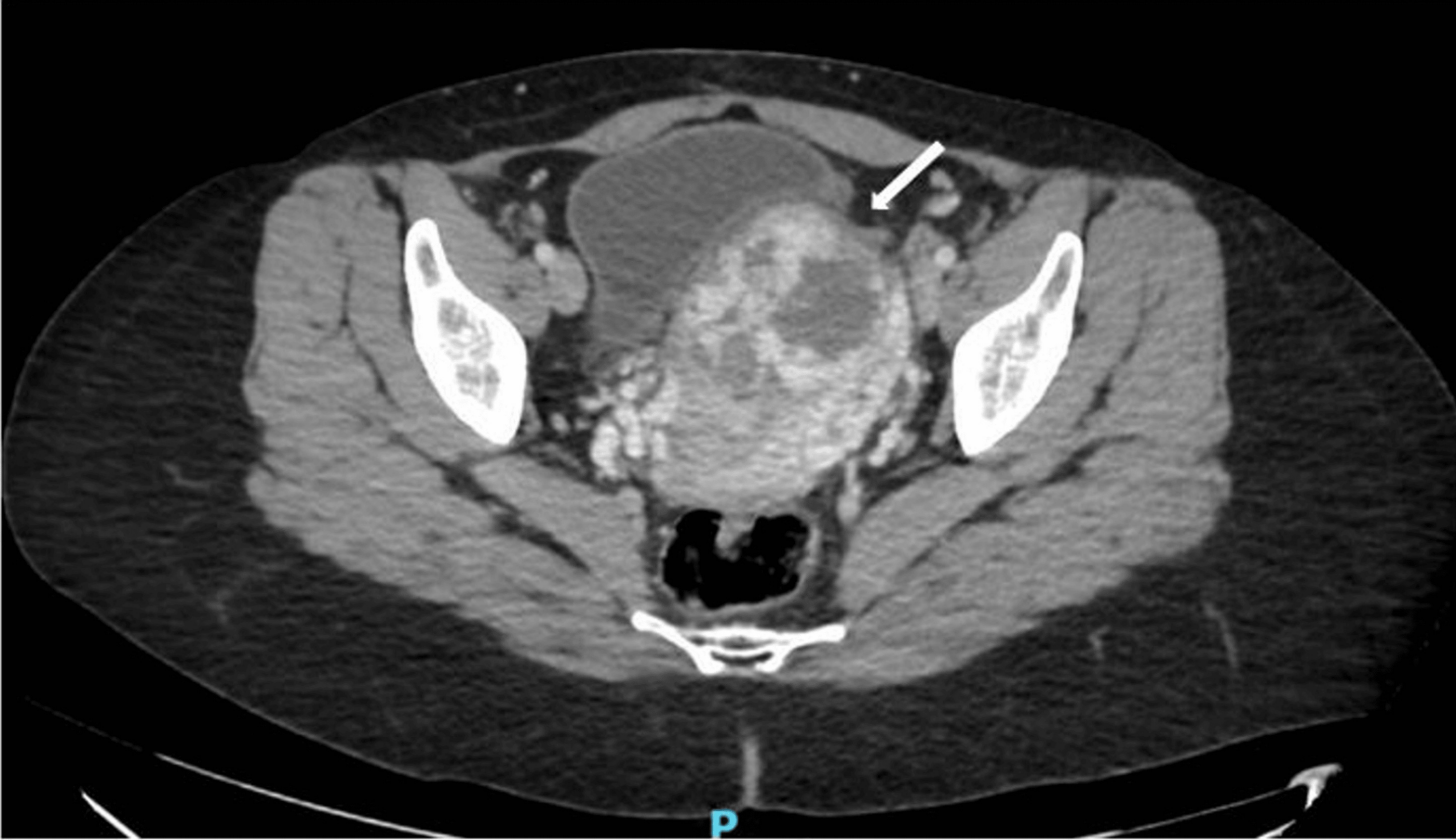
CT of the pelvis accentuates the low-lying nature of cesarean scar ectopic pregnancy and affirms the previously suspected imaging findings of a molar pregnancy. The arrow highlights the thin myometrium on the anterior uterus, raising concern for invasion into local structures and an elevated risk of uterine perforation, bladder injury, and hemorrhage if dilation and curettage were performed as the primary treatment modality.

**Figure 3:**
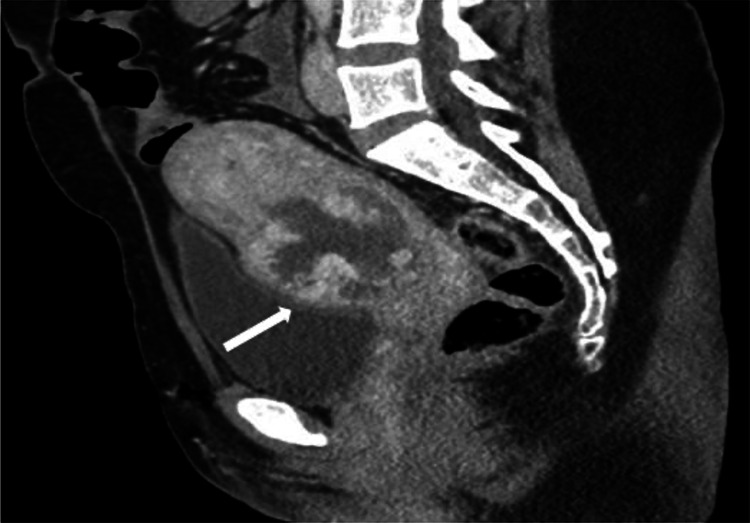
CT of sagittal section of the pelvis, highlighting the abnormal location and heterogeneity of the pregnancy. The arrow highlights the distinct uterine outpouching toward the bladder, which is common in cesarean scar ectopic pregnancies, and may indicate invasion into nearby structures.

Given the heightened suspicion of a CSEP and partial molar pregnancy, the patient underwent a chest X-ray in the ED to assess for metastatic gestational trophoblastic neoplasm (GTN), and a medial right upper lobe opacity was identified. A CT of the chest, abdomen, and pelvis was performed two days from the ED representation as an outpatient to further evaluate for invasion of the pregnancy into nearby structures, such as the bladder, and assess for metastatic disease (Figures 2, 3). The opacity previously seen on the chest radiograph corresponded to an indeterminate 1.7 cm sclerotic lesion on right clavicle, but the appearance was not consistent with GTN metastasis. No other lesions concerning for metastasis were identified. Uterine findings consisted of a heterogeneously enhancing mass in the lower uterine myometrium suspicious for cesarean scar location and molar pregnancy. A repeat b-hCG was collected two days later from the ED representation and remained stable at a value of 43,503 mIU/mL. 

Four days after obtaining the CT imaging, the patient’s history, imaging, and personal preferences for management were reviewed at a weekly gynecologic pre-operative conference. Given the concern for a possible CSEP complicated further by a partial molar pregnancy and the patient’s surgical history, the committee consensus was to recommend a gravid total abdominal hysterectomy (TAH), as the patient reported having satisfied their parity goals. Other treatment suggestions included a uterine artery embolization (UAE) before hysterectomy or dilation and curettage (D&C). The patient had reservations about undertaking another major abdominal surgery, as they are the sole caretaker of their young child. After counseling on the risks and benefits of all options, the patient elected to proceed with the conference committee's recommendation of a gravid TAH.

An uncomplicated gravid TAH was performed the same week as the patient’s preoperative conference. Surgical findings were notable for a gravid uterus with a ballooning lower uterine segment. The bladder was densely adherent to the lower uterine segment with large vessels bridging the bladder reflection. Despite these findings, on dissection there was no invasive involvement of the bladder by the placenta. The remainder of the uterus and fallopian tubes were normal in appearance. Total blood loss at time of the procedure was 850 mL. The patient had an unremarkable postoperative course and was discharged in stable condition on postoperative day two.

The patient postoperative progress was followed in the gynecologic oncology clinic. Serial b-hCG levels trended to a non-detectable level within a month of the procedure. A partial molar pregnancy was confirmed on pathology with cesarean scar involvement. No evidence of placenta accreta spectrum disorder was noted on final pathology report. Surveillance of the serum b-hCG was non-detectable at three- and six-month interval follow-up visits [1-2,5].

## Discussion

This case demonstrates the considerations for care of a notably rare combination of early pregnancy pathologies. It highlights the importance of close inspection of early pregnancy imaging, as the combination was initially missed at the initial presentation to an outside ED. The case also explores how to balance patient autonomy with careful counseling for highly morbid pregnancy complications.

Early identification of CSEPs relies on incorporating early imaging with a comprehensive obstetric history, including modes of delivery and pregnancy interval. Any low-lying pregnancy in the setting of a uterus with a previous cesarean scar should be evaluated for the involvement of the gestational sac, either extrinsic or extrinsic, as the findings are consistent with a CSEP and may impact delivery complications and treatment recommendations [4]. While many pieces of medical literature and committee opinions recommend early intervention to terminate a CSEP, some patients elect to proceed with expectant management [4,6,7]. Interestingly, a small retrospective study investigating pregnancy outcomes for extrinsic vs intrinsic CSEPs found that all pregnancies with intrinsic involvement and opted for expectant management had a hysterectomy at the time of delivery [6]. The counseling in our case of CSEP was difficult in the fact that a molar pregnancy remained on the differential due to the elevated b-hCG, as well as the hyperemic appearance and underdeveloped fetal pole.

Documentation of similar cases of molar CSEPs is exceedingly rare [3,7]. While a potential molar pregnancy diagnosis removes some of the burden from patients when considering expectant management or intervention, counseling may become more complicated. In both case series referenced, it was noted that almost all molar CSEPs, which were managed with hysteroscopy or D&C procedures, required additional emergent procedures, including UAE, repeat D&C, or hysterectomy secondary to substantial maternal hemorrhage. 

After a literature review and considering confounding conditions, both increasing the risk for significant maternal morbidity, our case was presented in a weekly group pre-operative conference. While all standard options for management were discussed, further evaluation via CT was critical in identifying the minimal anterior myometrium behind the bladder and bulge appearance. These findings may help classify the severity of CSEP and stratify risks based on procedures to be performed for treatment [3,6,7]. The committee ultimately recommend a gravid TAH, especially for a patient who does not desire future childbearing. Additional strategies considered included a pre-hysterectomy UAE to further reduce hemorrhage risk; however, there are limited reports on impacts on maternal outcomes. A UAE prior to an operative hysteroscopy or D&C is safe and may provide a reduction in morbidity for patients who may want fertility-sparing procedures. While a more robust trial is needed to make a comprehensive outcomes analysis, in a small retrospective study of patients desiring pregnancy after treatment of a CSEP with a UAE before a D&C, the investigators report to have found that 19 out of 23 patients (82.6%) were able to conceive again [8].

This case represents a positive outcome by reducing morbidity for a rare partial molar CSEP. While available literature on diagnosis and management is not robust, the emphasis on the higher morbidity of this combined pathology must be balanced with the patient's plan for future fertility. As similar cases of CSEP and molar pregnancies are reported, we hope the continued exploration into classification and management strategies may improve counseling and reduce maternal morbidity for such conditions [3,7].

## Conclusions

Close review of early pregnancy imaging is critical in making a diagnosis of a CSEP complicated by a molar pregnancy. The use of multiple imaging modalities may help narrow the differential diagnosis and evaluate the degree of invasion, ultimately helping to improve the counseling on management options. While there are reported cases of successfully performing less invasive procedures such as D&C to preserve fertility and treat CSEPs, a gravid hysterectomy could be offered as a treatment option for individuals with satisfied parity, as it may reduce the overall morbidity of this combination of rare pregnancy pathologies. 

## References

[1] Miller R, Gyamfi-Bannerman C (2022). Society for Maternal-Fetal Medicine Consult Series #63: cesarean scar ectopic pregnancy. Am J Obstet Gynecol.

[2] Jurkovic D, Hillaby K, Woelfer B, Lawrence A, Salim R, Elson CJ (2003). First-trimester diagnosis and management of pregnancies implanted into the lower uterine segment cesarean section scar. Ultrasound Obstet Gynecol.

[3] Al-Bataineh R, Rawashdeh S, Lataifeh LN, Alzghoul SM, Al Sharie AH, Obeidat R, Altal OF (2023). Cesarean scar ectopic partial molar pregnancy: a case report and a review of literature. Case Rep Womens Health.

[4] Ban Y, Shen J, Wang X (2023). Cesarean scar ectopic pregnancy clinical classification system with recommended surgical strategy. Obstet Gynecol.

[5] Soper JT (2021). Gestational trophoblastic disease: current evaluation and management. Obstet Gynecol.

[6] Kaelin Agten A, Cali G, Monteagudo A, Oviedo J, Ramos J, Timor-Tritsch I (2017). The clinical outcome of cesarean scar pregnancies implanted "on the scar" versus "in the niche". Am J Obstet Gynecol.

[7] Ling C, Zhao J, Qi X (2018). Partial molar pregnancy in the cesarean scar: a case report and literature review. Medicine (Baltimore).

[8] Chen YT, Chen YC, Chen M, Chang YJ, Yang SH, Tsai HD, Wu CH (2022). Reproductive outcomes of cesarean scar pregnancies treated with uterine artery embolization combined with curettage. Taiwan J Obstet Gynecol.

